# Editorial: The future of third molar surgery

**DOI:** 10.3389/froh.2024.1512305

**Published:** 2024-12-12

**Authors:** Ievgen I. Fesenko, João Luiz Gomes Carneiro Monteiro

**Affiliations:** ^1^ Department of Oral and Maxillofacial Surgery, Kyiv Medical University, Kyiv, Ukraine; ^2^ Department of Medicine, Brigham and Women’s Hospital and Harvard Medical School, Boston, MA, United States

**Keywords:** third molar, coronectomy, tooth transplantation, oral surgery, local anesthetic

**Editorial on the Research Topic**
The future of third molar surgery

The Research Topic, “*The Future of Third Molar Surgery*”, published in Frontiers, seeks to encourage clinicians and researchers to evaluate current trends in third molar research and reimagine the future of this field within oral and maxillofacial surgery. Historically, third molars have been associated with various pathological conditions (1–5). However, with technological advances and recent developments, specialists are now recognizing the potential of third molars beyond being a source of problems. They are exploring their value in several other areas—from replacing missing teeth through transplantation, to serving as an alternative source of autogenous bone via demineralized tooth chips, and even as a source of stem cells for tissue engineering (6–9).

Numerous articles, book chapters, and entire textbooks devoted to the surgery of third molars have already been published (10, 11). Nevertheless, less invasive procedures continue to develop, imaging and technologies are evolving, and research is being performed to even abolish third molar tooth buds during growth (12). This Research Topic consists of four peer-reviewed papers, three original studies, and one systematic review, involving 27 co-authors from multiple states. Lahoud et al. aimed to develop a simplified model of a transplanted tooth using the higher-order proper generalized decomposition method. Their efforts focused on understanding the biomechanics of transplanted teeth. This study represents a proof of concept, demonstrating the ability to provide real-time stress values through model order reduction techniques. Their results showed that the biomechanical behavior of a transplanted tooth, as simulated by finite element models, was accurately reproduced with a significant reduction in computing time. The authors suggest that future research explore how model order reduction could be applied to simulate complex occlusal schemes involving multiple dental contacts.

Pimenta et al. aimed to review systematic reviews which evaluated the effectiveness and safety of the preemptive use of anti-inflammatory and analgesic medicines in the management of postoperative pain, edema, and trismus in oral and maxillofacial surgery. Totally 19 studies were meticulously reviewed by authors. The effect of such drugs as betamethasone, dexamethasone, methylprednisolone, and prednisolone by different routes and likewise of celecoxib, diclofenac, etoricoxib, ibuprofen, ketorolac, meloxicam, nimesulide, and rofecoxib administered by oral, intramuscular, and intravenous routes were analyzed. According to the results, the above mentioned drugs were found to reduce pain, edema, and trismus in patients undergoing third molar surgery. Summarising the work, authors noted that further randomized clinical trials should be conducted to confirm these findings, given the wide variety of drugs, doses, and routes of administration used.

Poorna et al. concluded that the addition of 8 mg dexamethasone to 2 ml of 2% lignocaine in the nerve block before mandibular third molar surgery reduces the time of onset and significantly prolongs the duration of anesthesia with decreased swelling, trismus, and pain. Steroids mixed directly with the local anesthetic agent can minimize the post-operative sequelae associated with impacted third molar surgery with a single needle prick. The test group in their study included 35 patients. The paper is enhanced by pre- and post-operative clinical images showing the advantages of such a combination of drugs.

Gaballah et al. proposed a standardized protocol for third molar coronectomy involving standardized tooth sectioning parameters to minimize potential complications, surgical failure, and the need for further procedures. Their research was conducted on 69 eligible archived cone-beam computed tomograms. Drilling at a 25-degree angle to a depth of 9.5 mm is advisable to obtain the desired surgical results. The authors state that this approach will ensure no remaining enamel is left, minimize the chances of third molar root extrusion and future eruption, and improve outcomes.

Overall, this Research Topic highlights a predominant part of the research in third molar surgery. Each article will significantly strengthen this direction of surgery and create a foundation for further clinical implementations and scientific evaluations.
